# Upregulation of MiRNA-149-5p Reduces the Infract Volume in Middle Cerebral Artery Occlusion Rats by Modulating Cation-Chloride Cotransporters Expressions

**DOI:** 10.52547/ibj.3759

**Published:** 2022-11-08

**Authors:** Hossein Mostafavi, Narges Amoli, Elham Ghasemloo, Meysam Forouzandeh, Masoumeh Hosseini, Mehdi Eskandari

**Affiliations:** 1 Department of Physiology, School of Medicine, Zanjan University of Medical Sciences, Zanjan, Iran;; 2 Faculty of Life Sciences, Shahid-Beheshti University, Tehran, Iran;; 3 Laboratory Expert Depertment of Physiology, School of Medicine, Zanjan University of Medical Sciences, Zanjan, Iran

**Keywords:** Brain edama, Brain ischemia, Coenzyme Q10, MicroRNAs

## Abstract

**Introduction:**

Brain ischemia often leads to the chloride gradient alternations, which affects volume regulation and neuronal survival. Increase in NKCC1 expression and reduction in KCC2 level under ischemic condition results in inflammation and neuronal death. In this study, we investigated the effect of mimic miRNA and CoQ10 on the expression of CCCs (NKCC1 and KCC2) after cerebral ischemia.

**Methods:**

In this study, cerebral ischemia was modeled using the MCAO method. Rats were randomly divided into six groups: sham, model, NC, vehicle, and the first and second treatments. In the Sham group, ischemia was not induced, and no treatment was performed. In the Model group, ischemia induction was performed, and other groups, in addition to ischemia induction, received Scramble miRNA, Ethanol, mimic miRNA-149-5p and CoQ10, respectively. Each group was divided into three subgroups to assess the volume of the tissue damage and NDS in subgroup 1, brain water content in subgroup 2, level of miRNA-149-5p and CCC expressions in subgroup 3.

**Results:**

Our data suggested that the use of mimic miRNA and Q10 increased the level of miRNA-149 and KCC2 expression and decreased NDS, NKCC1 expression, brain water content, and infract volume.

**Conclusion:**

Findings of this study suggest that the mimic miRNA and Q10 may have neuroprotective effects through reducing infract volume and brain water content and modulating the expression of CCCS after brain ischemia.

## INTRODUCTION

Pathophysiology of ischemic stroke entails inflammation, atherosclerosis, blood coagulation, and platelet activation^[^^1^^]^. This condition involves disruption of the aerobic metabolism, which eventually leads to cellular death due to the decreased ATP levels and ionic imbalance across the neuronal cell membrane. Neuronal ischemia results in chloride gradient alterations, which influence the excitatory-inhibitory balance of neurons, volume infraction, and neuronal survival. Chloride homeostasis in the brain is primarily regulated by two CCCs: K^+^/Cl^-^ cotransporter KCC2, which extrudes Cl and K^+^, and Na^+^/K^+^/Cl^-^ cotransporter NKCC1, which shuttles Cl^-^, K^+^, and Na^+^ into the neuron. During the ischemic process, several changes occur in the expression and activity of CCCs, as well^[^^2^^]^. It has been shown that the mRNA expression of KCC2 in the rat brain is minimal at birth and increases in early postnatal life, while that of NKCC1 is the opposite at birth and in mature neurons. During neuron development, KCC2 expression is upregulated, while NKCC1 levels decrease^[^^3^^]^. 

Previous studies have shown that the profile of KCC2 and NKCC1 in neonatal neurons reappears in mature neurons under some pathophysiological conditions, such as brain ischemia^[^^4^^]^. In the early phase of cerebral ischemia, the level of intracellular Ca^++^ and extracellular K^+^ markedly increase in the ischemia area. Accordingly, with the activity of NKCC1, the transfer of sodium, potassium, and chlorine is modulated, which allows water to enter the cell and causes astrocyte swelling, cytotoxic edema, and neuronal infraction. Under ischemic challenges, NKCC1 causes excitotoxicity, excessive nitric oxide production, and cytokine (such as IL-1β, IL-6, and TNF-α) release, which later promotes BBB disruption in the ischemic area, resulting in the exacerbation of cerebral edema^[^^5^^]^. 

MiRNAs play important roles in physiological and pathological processes of neurodegenerative disorders and progression of certain neurological diseases, such as ischemic stroke. Several different miRNAs and their target genes have been recognized to be involved in the pathophysiology of ischemic stroke^[^^1^^]^. These noncoding RNA molecules are post-transcriptional regulators that bind specifically to the 3′-untranslated region of targeted mRNAs, giving rise to translational inhibition^[^^4^^]^. Among different miRNAs, miRNA-149-5p regulates BBB permeability by targeting S1PR2 of pericytes, which decreases BBB leakage. These data suggest that miRNA-149-5p may serve as a potential target for treating BBB disruption after ischemic stroke^[^^6^^]^. 

CoQ10 is an effective endogenously synthesized lipid and soluble antioxidant, which is a key component of the oxidative phosphorylation process in the mitochondria. The major form of CoQ10 found in the living organism is the reduced form, that is responsible for its antioxidant properties. Apart from its antioxidative function, CoQ10 appears to modulate immune functions by largely unknown mechanisms. It also decreases IL-1 and reduces the release of TNF-α in human and animal macrophages^[^^7^^]^. Chloride-cationic transporters have been shown to be potential therapeutic targets in neurological disorders; therefore, their dysfunction are involved in the secondary damage caused by cerebral edema (percentage of brain water) after cerebral ischemia^[^^8^^]^. The present study investigates the function of NKCC1 and KCC2 and the effect of mimic miRNA and CoQ10 on the expression of NKCC1 and KCC2. 

## MATERIALS AND METHODS

**Animal groups**

 Brain ischemia was modeled by MCAO. Male Wistar rats (n = 108) were randomly divided into six groups: Sham (without surgery and treatment), Model (MCAO), NC (MCAO + scramble miRNA), Vehicle (MCAO + Ethanol), First treatment: (MCAO + mimic miRNA), and Second treatment: (MCAO + Q10). Each group was divided into three subgroups to assess the volume of the tissue damage and NDS in subgroup 1, brain water content in subgroup 2, level of miRNA-149-5p and CCC expressions in subgroup 3.

**Animal preparation**

 For this study, 108 adult male Wistar rats (250–300 g) were obtained from the Pasteur Institute of Iran (Karaj). All the rats were kept in suitable living conditions with 12 h light/dark cycle at room temperature (22 ± 2 °C), appropriate humidity and free access to food and water throughout the study. 

**Establishment of MCAO model**

The MCAO model was established using adult male Wistar rats as described before^[^^9^^]^. In brief, the rats were anesthetized by intraperitoneal injection of ketamine (60 mg/kg) and xylazine (10 mg/kg) before the surgical procedure. After complete anesthesia, the animals were supine fixed on the operating table, the neck hair was clipped, and skin was disinfected with iodine volt gauze. The right common carotid artery of rats was fully exposed by blunt separation under a microscope. The internal and external carotid arteries were then separated. A poly-L-lysine-coated 3–0 monofilament nylon suture (403734PK5RE, Doccol Corporation, USA) with a rounded tip was introduced into the right internal carotid through the external carotid stump and advanced ~18 mm past the carotid bifurcation to occlude the origin of the middle cerebral artery. After placing in carotid for 1 h, the nylon thread was removed, and then perfusion was performed^[^^6^^]^. 

**Treatments of rats**

Rats were fixed in a stereotaxic apparatus after being anesthetized intraperitoneally with ketamine (60 mg/kg) and xylazine (10 mg/kg). Mimic miRNA/first treatment group (5 µl with a concentration of 2.5 nmol, Thermo Fisher, USA) and scramble149-5p/NC group (2.5 nmol/L) were injected into the right lateral ventricle of rats (coordinates: 1 mm caudal to bregma, 2.5 mm lateral to midline, and 3.5 mm deep from the surface of skull) at the rate of 1 µl/min^[^^6^^]^. Thirty minutes after injection, the rats received MCAO induction. CoQ10/second treatment group (30 mg/kg) and its solvent (Ethanol)/Vehicle group (10 mg/kg) were injected 45 minutes after MCAO induction via caudate vein. Rats were then returned to recovery cages and closely observed until complete recovery from anesthesia.

**Neurological deficits score**

Twenty four hours after induced ischemia, assessment of motor and neurological deficits were conducted based on a previously described scale^[^^10^^]^. The results of this assay were scored on a scale from 0 to 5. Animals scored as zero showed no motor neurological deficits. A complete deficit failure in the distal part of forepaws on contralateral side was considered a weak impairment and scored one. Animals with left rotation sign scored two and categorized as moderate deficit. Falling on left side is a severe impairment and scored as three. Score number four belonged to the animals with low level of consciousness, i.e. incapable of moving spontaneously. Score five was assigned to animal’s death, specifically due to brain stroke 24 hours after surgery with severe brain injury after staining with TTC.

**TTC staining for morphological examination**

Brain was first removed under anesthesia and placed at -20 °C for 30 min, then sliced transversely from anterior to posterior extremity with same thickness and finally stained with 2% TTC. The images of brain sections were captured and then analyzed by an USB Image tool (v. 1.83.0). The quantitative analysis of infarct volume was performed by its comparison with contralateral brain volume using the calculation formula as follows: Tissue damage volume = (left hemisphere area × 2) - [(right hemisphere area × 2) - (damaged area × 2)]^[^^11^^]^. 

**Measurement of the brain** **water content**

Twenty-four hours after brain ischemia induction, all the rats were killed using a high dose of anesthetic drug, and then the animals’ brains were removed. The cerebellum, pons, and olfactory bulbs were isolated, and the net weight of brain hemispheres (WW) was measured. The brain hemispheres were placed in an oven with 120 °C temperature for 24 hours, and the dry weight (DW) was measured. Finally, the brain water content was calculated according to the formula: ([WW-DW]/WW) × 100.

**Examining the expression of genes by qRT-PCR**

Total RNA was isolated using TRIzol reagent, and 5 mg of the solution was used to synthesize cDNA using the mRNA/miRNA cDNA Synthesis Kit (BON209002 Research Center, Iran). cDNA was amplified with the SYBR Premix Ex TaqTM Kit (RR420A, USA). Real-time qRT-PCR was performed with a thermocycler according to the manufacturer’s recommendations. The forward and reverse primers for NKCC1 and KCC2 and also the forward primer for miRNA-149-5p were provided in Table1, and the common reverse primer sequence was provided by the mRNA/miRNA cDNA Synthesis Kit (BON209002 Research Center) (Table1). Data were analyzed using the comparative Ct method (2^-ΔΔCt^) and normalized to β-actin (for NKCC1 and KCC2) or Snord (for miRNAs)^[^^11^^]^. 

**Statistical analysis**

Data were shown as mean ± SEM. Statistical analysis was performed using SPSS Statistics 24 and Excel. The one-way ANOVA of Fisher’s least significant difference test and Tukey’s multiple range test were used. The data obtained from the study of neurological deficits were analyzed by Kruskal-Wallis test. A value of *p* < 0.05 was considered statistically significant.

**Table 1:** Primer sequence for RT-qPCR

**Genes**	**Primer sequences**
*NKCC1*	Forward: 5'-TCACGAACACCGCAGCATCA-3' Reverse: 5'-TCTATCAGTGGAGCAACGTG-3'
	
*KCC2*	Forward: 5'- AGGTGGAAGTCGTGGAGATG-3' Reverse: 5'-TGAGGTGCATCTGTTTGAGG-3'
	
*β-actin*	Forward: 5'-GCTCTGGCTCCTAGCACCAT-3' Reverse: 5'-GCCACCGATCCACACAGAGT-3'
	
*miRNA-149-5p*	Forward: 5'-TCTGGCTCCGTGTCTTCACTCCC-3' Common reverse primer in BON microRNA QPCR Master mix kit

**Fig. 1:**
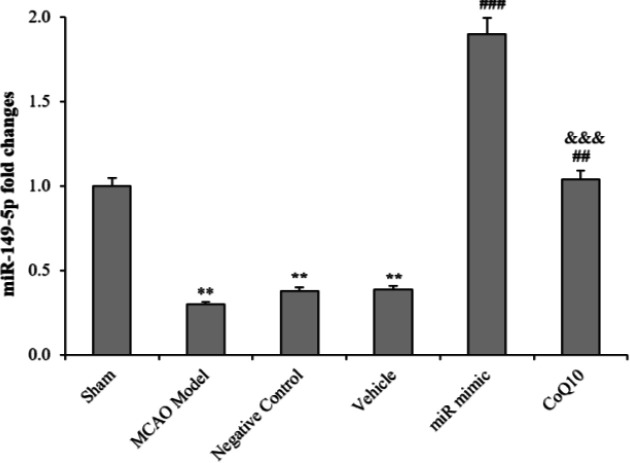
Changes of miRNA-149-5p following cerebral ischemia and treatment with mimic miRNA and CoQ10. The data are expressed as the mean ± SEM. ^**^p < 0.01 compared to Sham Group; ^##^*p *< 0.01 compared to MCAO Model, NC, and Vehicle groups; ^###^*p* < 0.001 compared to MCAO Model, NC and Vehicle groups; ^&&&^*p* < 0.001 compared to mimic miRNA group (n = 6)

## RESULTS

**Increased level of miRNA-149-5p in brain after treatment**

The level of miRNA-149-5p in MCAO rats was detected by real-time qRT-PCR. The result showed a decreased level of miRNA-149-5p after MCAO in the MCAO model group and a significant difference was observed between Sham and MCAO model groups (*p *< 0.01). Treatment with mimic miRNA and CoQ10 led to an increased level of miRNA-149-5p, suggesting a meaningful rise in the first and second treatment groups compared to MCAO model group (*p < *0.001). Results indicated that using scramble miRNA and ethanol in NC and vehicle groups, respectively, had no effect on the level of miRNA-149-5p, and no significant difference was found between the NC and vehicle groups compared to the Model group. Also, the level of miRNA-149-5p in the first treatment group showed a significant increase compared to the second treatment group (*p* < 0.001), displaying that the use of mimic miRNA is more effective than CoQ10 in raising the level of miRNA-149-5p (Fig. 1).

**Fig. 2:**
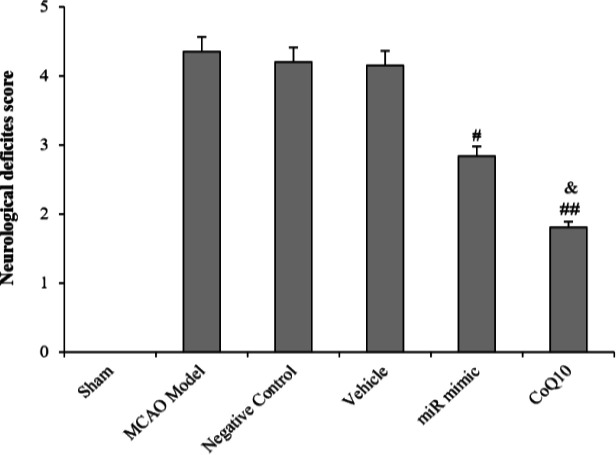
Evaluation of NDS following cerebral ischemia and treatment with mimic miRNA and CoQ10. The data are expressed as the mean ± SEM. ^#^*p *< 0.05 compared to MCAO Model, NC, and vehicle groups; ^##^*p *< 0.01 compared to MCAO Model, NC, and Vehicle groups; ^&^*p* < 0.05 compared to mimic miRNA group (n = 6).

**Reduction of neurological defects in treatment groups**

Statistical analysis of the neurological defects showed that brain ischemia induction in the studied rats led to motor disorders, while intraventricular injection of mimic miRNA and intravenous injection of CoQ10 resulted in a decrease in the score of neurological defects in the first (*p *< 0.05) and second (*p *< 0.01) treatment groups as compared to the MCAO group. There was no significant difference among the MCAO, NC, and vehicle groups, which indicates that scramble miRNA and ethanol had no positive effect on NDS. The score of neurological defects in CoQ10-treated group, in comparison to mimic miRNA-treated group, showed a meaningful decrease (*p *< 0.05) decline (Fig. 2).

**Reduction of NKCC1 expression after treatment**

**Fig. 3:**
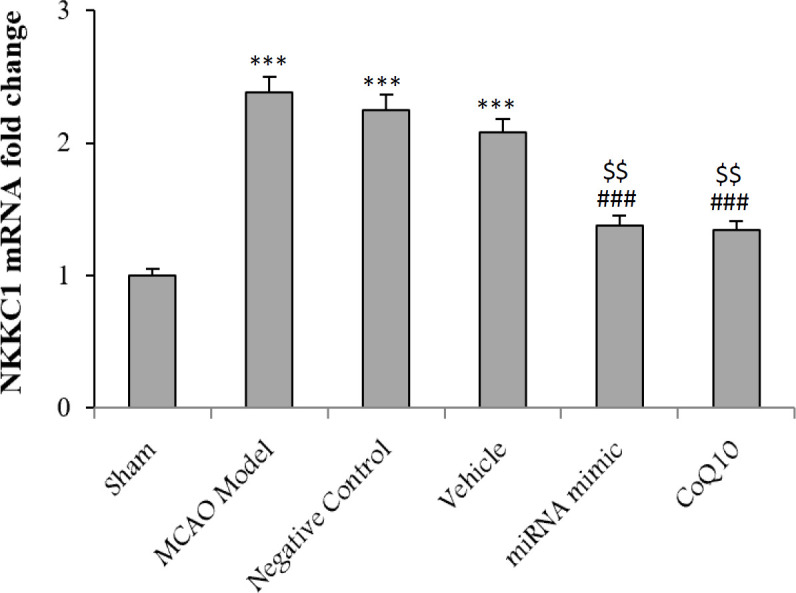
NKCC1 changes following cerebral ischemia and treatment with mimic miRNA and CoQ10. The data are expressed as the mean ± SEM. ^***^p < 0.001 compared to Sham group; ^###^*p* < 0.001 compared to MCAO Model group; ^$$^*p* < 0.01 compared to NC and Vehicle groups (n = 6)

The results revealed an increase in the level of NKCC1 expression in the Model group compared to sham after ischemia (*p *< 0.001). Intraventricular injection of mimic miRNA and CoQ10 decreased NKCC1 expression (*p *< 0.001). The difference was significant between the first and second treatment groups relative to the MCAO Model group (*p *< 0.01) (Fig. 3). The level of NKCC1 expression remained constant after injection of scramble miRNA and ethanol.

**Increased level of KCC2 expression after treatment**

**Fig. 4:**
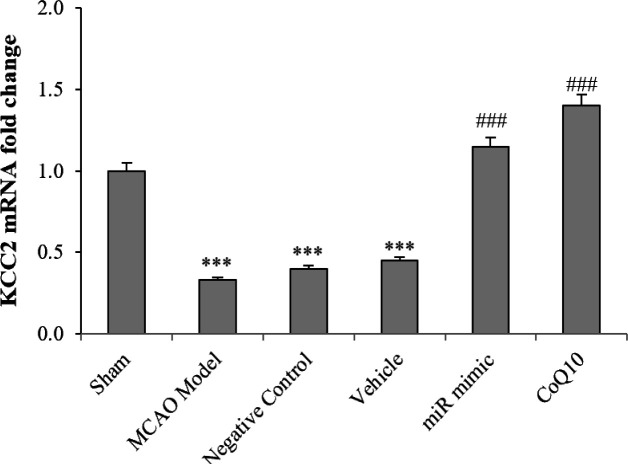
Changes of KCC2 following cerebral ischemia and treatment with mimic miRNA and CoQ10. The data are expressed as the mean ± SEM. ^***^p < 0.001 compared to sham group; ^###^*p* < 0.001 compared to MCAO Model, NC, and vehicle groups (n = 6)

Results of the real-time qRT-PCR indicated a reduction in the level of KCC2 expression due to brain ischemia. A significant reduction was also observed in the level of KCC2 expression in the MCAO Model group compared to the sham group (*p *< 0.001). Treatment with mimic miRNA and CoQ10 increased KCC2 expression, leading to a significant rise in the KCC2 level compared to the MCAO Model group (*p < *0.001). Scramble miRNA and ethanol injection had no effect on the KCC2 level in NC and vehicle groups compared to the Model group (Fig. 4).

**Reduction in the volume of tissue damage after treatment**

**Fig. 5:**
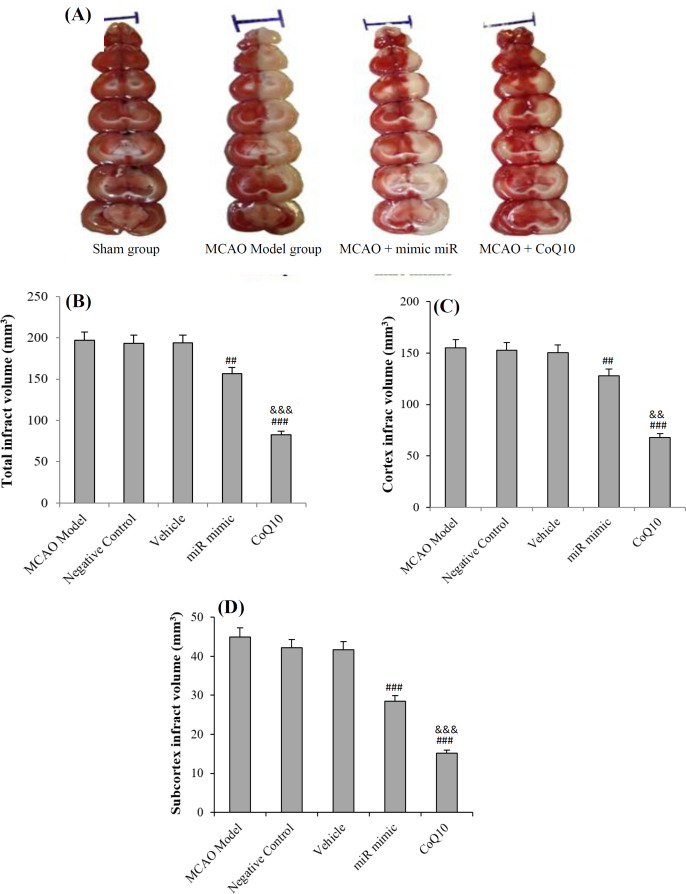
Sample of coronal sections of rat brain stained with 2% TTC solution (A) and changes in the volume of tissue damage in studied groups in cortical and subcortical regions (B-D). The red and white areas indicate the healthy and damaged areas, respectively. In sham group rats, no white area is visible, while in the Model rats, almost half of the sections are white. Treatment with mimic miRNA and CoQ10 resulted in a decrease in white areas compared to the MCAO Model group. The lines above the images indicate a length of 1 cm. The data are expressed as the mean ± SEM. ^##^*p* < 0.01 compared to MCAO Model, NC, and Vehicle groups; ^###^*p* < 0.001 compared to MCAO Model, NC, and Vehicle groups; ^&&^*p* < 0.01) compared to mimic miRNA group; ^&&&^*p* < 0.001 compared to mimic miRNA group (n = 6)

The volume of the damaged tissue was evaluated by staining with TTC solution (Fig. 5A), and the total tissue damage volume (Fig. 5B) and the volume of tissue damage in the cortical (Fig. 5C) and subcortical areas (Fig. 5D) were measured. Data analysis showed that cerebral ischemia led to tissue damage in the affected hemisphere. Intraventricular injection of mimic miRNA in the first treatment group and intravenous injection of CoQ10 in the second treatment group resulted in a significant decrease in the total volume of the damaged tissue (*p *< 0.001), as well as the volume of tissue damage in cortical (*p *< 0.05; Fig. 5B) and subcortical (*p *< 0.05; Fig. 5D) areas. Also, a meaningful difference was found in the volume of total tissue damage and tissue damage volume in cortex and subcortical regions between treatment groups and the MCAO group. It was found that Q10 was more effective than mimic miRNA in reducing the total tissue damage and tissue damage volumes in cortex and subcortical areas (Fig. 5B-5D). There was no significant difference between the volume of tissue damage in the NC, MCAO, and vehicle groups. Moreover, scramble miRNA and ethanol had no effect on the reduction of volume of tissue damage (Fig. 5B-5D).

**Reduction of brain water content after treatment**

**Fig. 6:**
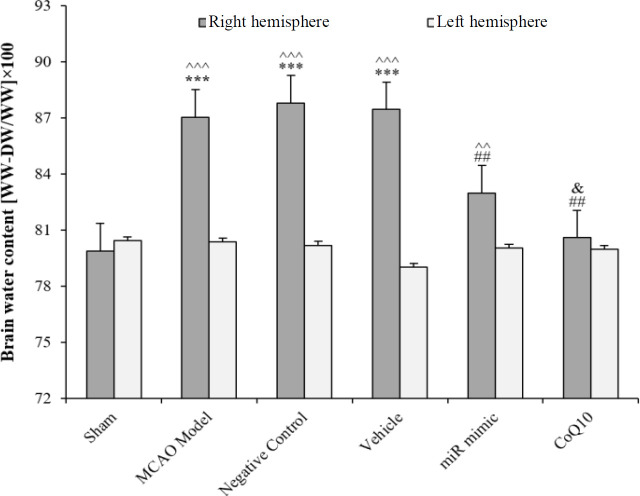
Changes in the brain water content of the damaged (right) and healthy (left) hemispheres of rats in the studied groups. The data are expressed as the mean ± SEM. ^***^p < 0.001 compared to sham group; ^##^*p* < 0.01 compared to MCAO Model, NC, and Vehicle groups; ^&^*p* < 0.1 compared to mimic miRNA group; ^^^^^*p* < 0.001 and ^^^^*p* < 0.01 compared to opposite hemisphere in the same group (n = 6)

Induction of cerebral ischemia by the MCAO method led to an increase in the brain water content in the damaged hemisphere (right), and a significant difference was observed in terms of the brain water content between the right and left hemisphere of the MCAO Model group (*p *< 0.001). The use of Q10 solvent and scramble miRNA in vehicle and NC groups did not cause any significant change in the brain water content of the damaged hemisphere, and there was also no significant difference between the brain water content of damaged hemispheres in the vehicle and NC groups. Brain water content of the damaged hemisphere in groups receiving mimic miRNA (*p *< 0.001) and CoQ10 (*p *< 0.001) had a significant decrease compared to the right hemisphere of the MCAO Model group. This reduction was more evident in the group receiving CoQ10 because the percentage of brain water content in the right hemispheres of the first and second treatment groups was significantly different (*p *< 0.001). However, there was no significant difference between the right and left hemispheres of all groups relative to each other (Fig. 6).

## DISCUSSION

The results of this study showed downregulation of miRNA-149-5p and KCC2 and upregulation of NKCC1 following cerebral ischemia. The study also suggested that treatment with mimic miRNA and Q10 led to the upregulation of miRNA-149-5p and KCC2 and downregulation of KCC1. Overall, there was a reduction in the volume of the tissue damage, brain water content, and neurological deficits as consequences of brain ischemia. Previous studies have demonstrated that the level of miRNA-149-5p decreases after neurological disorders, including brain ischemia^[^^11^^]^ and Parkinson’s disease^[^^12^^]^. In addition, the use of mimic miRNA upregulated miRNA-149-5p level, which reduced neuronal damage and improved complications of the mentioned disease. These findings are in the same direction with the result of the present study. 

Q10 is capable of modulating the gene expression through its effect on different factors involved in the expression of miRNA genes^[^^13^^,^^14^^]^. For instance, Q10 has been shown to regulate *miRNA-378* gene expression by affecting the factors involved in the expression of this gene^[^^15^^]^. In the present study, injection of the mimic miRNA and Q10 led to the increased level of the miRNA-149-5p and reduced brain ischemia outcomes. On the other hand, NKCC1 expression elevated following cerebral ischemia, which is associated with an elevation in the consequences of brain ischemia, including neuronal damage. The inhibition of this transporter resulting in a reduction in neuronal damage^[^^16^^]^ has a protective effect on neurons^[^^17^^]^. It is also revealed that the increased NKCC1 expression level resulted in the neuronal damage by eliminating ionic homeostasis. Moreover, its inhibition by bumetanide reduced the extent of ischemic damage, as well as cerebral edema^[^^18^^]^. In fact, cerebral ischemia gives rise to NKCC1 phosphorylation by activating WNK/SPAK/OSR1 pathway and increases its activity, and inhibition of NKCC1 can reduce tissue damage, neuronal death, edema, and neurological defects^[^^19^^]^. Studies have implied that inflammatory factors such as IL-6, TNF-α are targets of miRNA-149-5p^[^^11^^,^^12^^]^, and the miRNA improves BBB function and integrity by reducing the mentioned factors following cerebral ischemia and decreases ischemia-reperfusion injury^[^^11^^]^. It has been found that the antioxidant effect of Q10 and the reduction of free radical production^[^^20^^]^ after brain ischemia can decease inflammation and damage the binding proteins in the tight junctions such as ZO-1 and occluding in BBB^[^^11^^]^. Furthermore, CoQ10 reduced cerebral infarct volumes by 67% through its effect on the neuronal death pathways and enhanced sensory-motor functions in rats^[^^21^^]^. Since it is specified that increased glutamate and cytokines are involved in stimulating NKCC1 activity following cerebral ischemia^[^^22^^]^, it seems that the effect of miRNA-149-5p and CoQ10 on inflammatory pathways and subsequently reduction of the inflammatory factors and oxidative stress decreases phosphorylation and expression of NKCC1, thereby reducing the risk of cerebral ischemia, including neuronal damage. Inhibition of NKCC1 by specific inhibitors has been shown to mitigate approximately half the volume of the tissue damage following cerebral ischemia and also improve sensory-motor function and reduce mortality^[^^19^^]^. MiRNA-101 facilitates GABAergic switching by targeting NKCC^[^^23^^]^. Therefore, it can be deduced that miRNA-149-5p and CoQ10 decrease NKCC1 expression by reducing inflammation and oxidative stress, consequently lower the volume of the tissue damage, brain water content, and neurological defects. However, more further studies are needed to reveal the exact mechanism of the effect of MiRNA and CoQ10 on reducing tissue damage. In mature neurons, upregulation of KCC2 expression leads to the modulation of ion homeostasis. However, KCC2 level expression decreases in a number of neurological disorders such as ischemic stroke^[^^24^^]^. In neonatal rats exposing to hypoxia, a meaningful decrease occurred in the KCC2 protein level in the cortex 24 hours post injury. In this regard, it has also been exhibited that hypoxia-ischemia in the neonatal rats was associated with KCC2 loss in hippocampus and cortex^[^^25^^]^. Furthermore, it was explored that the expression of KCC2 in motor neurons reduced by 85% after brain ischemia, and upregulation of this transporter decreased hemiplegia and neuronal damage^[26]^. Increased phosphorylation and expression of KCC2 improves neurological defects and cognitive impairments following ischemia-reperfusion injury^[^^27^^]^. The present study, similar to the above-mentioned studies^[^^22^^-^^27^^]^, showed that KCC2 level expression decreased after brain ischemia, and use of mimic miRNA and CoQ10 upregulated this transporter, which is associated with a reduction in neurological deficits and volume of tissue damage. 

Earlier studies have revealed that elevating the level of miRNA-149-5p after the use of mimic miRNA increases BBB integrity and reduces brain ischemia infraction volume by influencing the effective factors in pericytes migration^[^^28^^]^. MiRNA-149-5p also affects MMP-9 and superoxide dismutase in pericytes and improves BBB function following cerebral ischemia-reperfusion^[^^29^^]^. However, the intravenous injection of CoQ10 decreases mortality in rats after cerebral ischemia and reduces the volume of tissue damage and neurological defects^[^^30^^]^. CoQ10 can also improve neuronal functions and reduce the volume of tissue damage by affecting inflammatory cytokines and key factors on neuronal death^[^^31^^]^. Therefore, it seems that the effect of miR149-5p and CoQ10 on increasing KCC2 expression is associated with a reduction in the infraction volume and NDS of cerebral ischemia. Therefore, it can be concluded that treatment with mimic miR and Q10 prevents BBB damage and inflammatory factor production by increasing the expression of KCC2. 

One of the results of this study was the stronger neuroprotective effect of CoQ10 than miRNA149-5p, which is likely due to the reduced volume of tissue damage. It appears that Q10 not only downregulates NKCC1, upregulates KCC2 and reduces damage to BBB and NDS, but also has a synergic effect on preventing the spread of ischemic consequences by increasing miRNA149-5p level expression. Therefore, it seems that multiple pathways activated by the antioxidant and anti-inflammatory effects of Q10 lead to the amplification of its neuroprotective effects compared to miR-149-5p. A limitation of this study is the lack of identification of the exact mechanism that Q10 causes an increase in the level of miRNA-149-5p. Further studies are needed to identify the exact molecular process in the regulation of miRNA-149-5p expression by Q10.

## DECLARATIONS

### Acknowledgments

This article is extracted from the master's degree of Narges Amoli, a student of Zanjan University of Medical Sciences, Zanjan, Iran.

### Ethical statement

The above-mentioned sampling protocols were approved by the Institutional Guidelines of the Animal Care and Use Committee, Zanjan University of Medical Sciences, Zanjan, Iran (Ethical no: IR.ZUMS.REC. 1400.004). 

### Data availability

The analyzed data sets generated during the study are available from the corresponding author on reasonable request.

### Author contributions

HM: conceptualization, methodology, data curation, software, validation, writing, reviewing, investigation, project administration, and supervision; NA: data curation, software, validation, writing, reviewing, and investigation; EG: conceptualization, methodology, software, validation, writing, reviewing, and resources; MF: methodology, writing, original draft preparation, and editing; MH: data curation, software, validation, writing, and reviewing; ME: software and validation.

### Conflict of interest

None declared.

### Funding/support

This study was supported by grant No: A-12-871-11 from Deputy of Research and Technology, Zanjan University of Medical Sciences, Zanjan, Iran.
